# Abnormal psychological performance as potential marker for high risk of internet gaming disorder: An eye-tracking study and support vector machine analysis

**DOI:** 10.3389/fpsyg.2022.995918

**Published:** 2022-09-16

**Authors:** Shuai Wang, Jialing Li, Siyu Wang, Wei Wang, Can Mi, Wenjing Xiong, Zhengjia Xu, Longxing Tang, Yanzhang Li

**Affiliations:** ^1^School of Psychology, Chengdu Medical College, Chengdu, China; ^2^School of Clinical Medicine, Chengdu Medical College, Chengdu, China

**Keywords:** high risk of internet gaming disorder, impulse control, emotion regulation, response inhibition, marker

## Abstract

Individuals with high risk of internet gaming disorder (HIGD) showed abnormal psychological performances in response inhibition, impulse control, and emotion regulation, and are considered the high-risk stage of internet gaming disorder (IGD). The identification of this population mainly relies on clinical scales, which are less accurate. This study aimed to explore whether these performances have highly accurate for discriminating HIGD from low-risk ones. Eye tracking based anti-saccade task, Barratt impulsiveness scale (BIS), and Wong and Law emotional intelligence scale (WLEIS) were used to evaluate psychological performances in 57 individuals with HIGD and 52 matched low risk of internet gaming disorder (LIGD). HIGD group showed significantly increased BIS total (*t* = −2.875, *p =* 0.005), attention (*t* = −2.139, *p =* 0.035), motor (*t* = −2.017, *p =* 0.046), and non-planning (*t* = −2.171, *p =* 0.032) scores, but significantly decreased WLEIS emotion regulation score (*t* = 2.636, *p =* 0.010) and correct rate of eye tracking anti-saccade task (*t* = 2.294, *p =* 0.024) compared with LIGD group. BIS total score was negatively correlated with the WLEIS total (*r* = −0.473, *p <* 0.001) and WLEIS emotion regulation (*r* = −0.366, *p <* 0.001) scores. A combination of the WLEIS emotion regulation score and the correct rate of anti-saccade task could discriminate HIGD from LIGD with 91.23% sensitivity, 82.69% specificity, and 87.16% accuracy. Participants with higher gaming hours daily were 40 times more likely to be high risk than their counterparts (*p <* 0.001). Hence, psychological performances were worse in HIGD. A combination of abnormal emotion regulation and response inhibition might be a potential marker to identify HIGD individuals.

## Introduction

Following the inclusion of “internet gaming disorder (IGD)” in the fifth edition of Diagnostic and Statistical Manual of Mental Disorders (DSM-5), the 11th edition of the International Classification of Diseases (ICD-11) was approved, also included “gaming disorder (GD)” as a new disease (Petry and O'Brien, 2013; Ko et al., 2020). GD is mainly divided into online and offline participation forms, and online is IGD, which is a hot research topic at present. IGD is characterized by uncontrol of gaming, lasting at least 12 months (Jo et al., 2019). Patients with IGD generally seldom or do not engage in other activities, such as social interaction, study, and work. Their everyday life rhythms are also seriously affected. The prevalence of game-related problems in China is 3.5–17.0%, and the average prevalence of GD is about 5.0% (Chiang et al., 2021). IGD brings a huge burden and harm to society and the patient’s family. Therefore, early screening and intervention of IGD are essential, which could significantly reduce the incidence risk.

Individuals with IGD typically progress from low to high risk (Wartberg et al., 2019). Questionnaires or scales, such as the Internet Gaming Disorder Questionnaire (IGDQ) and nine-item Internet Gaming Disorder Scale-Short Form (IGDS9-SF), are often used in clinical practice to assess the risk status and symptom severity (Jeromin et al., 2016; Qin et al., 2020). High risk of IGD (HIGD) is defined as gaming participants with an IGDQ score of more than five and a Chen Internet Addiction Scale (CIAS) score of more than 63 or exceeding the threshold of other scales (Ji and Hsiao, 2020; Jeong et al., 2021). The remaining gaming participants are defined as low risk of IGD (LIGD). A previous study has shown that HIGD individuals exhibit abnormal behaviors and instantaneous frequency distribution of respiratory signals (Ji and Hsiao, 2020). Instantaneous frequency distribution of respiratory signals and structural and functional magnetic resonance imaging were attempted to identify HIGD or IGD (Ji and Hsiao, 2020; Han et al., 2021). However, its cost is relatively high, and its accuracy is uncertain. In addition, the questionnaire is the primary method of classifying HIGD and LIGD (Lai et al., 2021). However, the results obtained using questionnaires and scales mainly reflect the game-related behavioral and psychological changes and might be affected by several factors (Lai et al., 2021). So, it is necessary to identify HIGD with some stable indicators that could reflect the overall changes of the individual.

Multiple behavioral and psychological functions, such as response inhibition, impulse control, and emotional regulation, were found to be abnormalities in IGD. Response inhibition is a cognitive process that permits individuals to inhibit their habitual, impulses, or dominant behavior to complete their original goals (Ilieva et al., 2015). A cross-section study evaluated the brain correlates of response inhibition in patients with IGD and showed that patients with IGD had lower activity of the right supplement motor area (SMA)/pre-SMA, suggesting patients with IGD had impaired response inhibition (Chen et al., 2015). Impulse control issues refer to people’s difficulty in stopping themselves from engaging in certain behaviors. Patients with IGD have deficits in impulse control (Wang et al., 2017). A positive association between impulsivity and IGD was identified by a meta-analysis that included 32 studies (Şalvarlı and Griffiths, 2022). Emotion regulation is defined by the set of cognitive processes that influence emotional responses and is a complex process that encompasses the initiation, inhibition, or modulation of emotional functioning (Lin et al., 2020). A cross-section study revealed that emotion adjustment scores in IGD individuals were significantly lower than in controls (Lin et al., 2020). Indeed, subjects with IGD were more likely to suppress their emotions (Yen et al., 2017). Poor response inhibition may constitute one facet of impulsivity (Wilbertz et al., 2014). In patients with schizophrenia that poorer impulse control was associated with greater activation within the right ventrolateral prefrontal cortex during response inhibition (Kaladjian et al., 2011). Further, worse emotional regulation also predicted worse response inhibition (King, 2020). High emotion dysregulation scored significantly higher on self-report measures of impulsivity (Schreiber et al., 2012). So, the relationship among these three indicators (response inhibition, impulse control, and emotional regulation) was relatively clear, which implies a potential value for judging the risk of IGD.

Our recent study found that HIGD with escapism-based motivation had a higher Barratt impulsiveness score, lower abilities of emotion management, and worse response inhibition than controls (Wang et al., 2022). However, the differences in these psychological performances between HIGD and LIGD are still unclear. Thus, in the present study, we hypothesize that HIGD individuals might exhibit more abnormal impulse control, emotion regulation, and response inhibition than LIGD. A case–control design was performed on individuals with HIGD or LIGD. Furthermore, we also explored whether these abnormal psychological performances can be considered a potential marker for discriminating HIGD from LIGD with acceptable accuracy, stability, and expense.

## Materials and methods

### Participants

Individuals were screened from a cohort of 789 individuals, which was described in our previous report. The participant should be excluded if he or she was left-handed or suffered from a severe medical disorder, mental disorder, and substance abuse. According to sample size analysis (Li and Fine, 2004), this study must recruit at least 98 individuals overall to have 90% power with 5% type 1 error level and 10% loss to follow up to detect a 0.8 ± 0.1 expected sensitivity and 0.8 ± 0.1 expected specificity in the receiver operating characteristic curve (ROC) or support vector machine (SVM) analysis. In this study, we recruited 57 HIGD and 52 LIGD individuals. HIGD was defined as a gaming participant with an IGDQ score of ≥5 and a CIAS score of ≥64 (Ji and Hsiao, 2020). LIGD was defined as a gaming participant with an IGDQ score of <5 or a CIAS score of <64.

The study was conducted in accordance with the Helsinki Declaration. The Ethics Committee of Chengdu Medical College approved the study. All participants signed the written informed consent.

### General information and internet gaming behavior assessment

The participants’ basic information regarding love experience, upbringing, academic performance, residence, and demographics, was recorded in a self-administered questionnaire. Gaming information such as daily gaming hours, primary gaming type, and gaming history was also collected. The risk of IGD was assessed through both IGDQ and CIAS by two experienced clinicians.

### Psychological assessments

Barratt impulsiveness scale in the 11th (BIS; Yao et al., 2007) with 0.85 Kappa coefficient and 0.76 Cronbach’s αand the Wong and Law Emotional Intelligence Scale (WLEIS; Di et al., 2022) with 0.78 Kappa coefficient and 0.86 Cronbach’s α were used to measure the impulse control and emotion regulation.

Anti-saccade task-based eye-tracking test was used to measure their ability of response inhibition. The EyeLink 1000 system (SR Research Ltd., Canada) was used for eye-tracking tests (Wang et al., 2022). A sampling rate of 1,000 Hz recorded the movement of the dominant eye. The distance from the eyes to the computer monitor (1,024 × 768 resolutions, 60 Hz refresh rate) was set as 50 cm. Experiment Builder software (SR Research Ltd., Canada) was used to perform the procedure. The pro- and anti-saccade trials were designed for the anti-saccade task (Wang et al., 2022). The primary outcome was direction correct rate, and the secondary outcomes were fixation count, correct reaction time, and saccade count (Petry and O'Brien, 2013).

### Statistical analysis

The eye-tracking test data were extracted using DataViewer (SR Research Ltd., Canada). The general information, internet gaming behavior, and psychological assessment data were manually extracted from the original questionnaires. All the statistical analyses were performed using SPSS 22.0 (IBM Co., United States). To examine the differences in the demographic, internet gaming behavioral, and psychological indicators data between the groups, *X*^2^ and *t*-tests were used for the categorical and continuous variables, respectively. Once the significantly different values of psychological indicators between LIGD and HIGD were observed, the ROC analysis was used to detect these indicators’ specificity, sensitivity, and accuracy to discriminate HIGD from LIGD. LIBSVM software package^1^ (Chang and Lin, 2011), which can run in MATLAB (version R2015b, The MathWorks, Inc., USA), was used to perform SVM analysis to investigate the specificity, sensitivity, and accuracy of a combination of these indicators for identifying the HIGD. We included 57 HIGD individuals and 52 LIGD individuals in this analysis. Parameter optimization adopted the Grid search method and Gaussian radial basis function kernels. The “leave-one-out” cross-validation was applied to obtain the highest accuracy (Wang et al., 2016). Logistic regression was performed further to obtain the associated factors in the HIGD participants, and *p <* 0.05 results were considered statistically significant.

## Results

### Basic information of the study sample

The basic information of the sample is detailed in Table 1. In a comparison of age, gender, smoking history, academic performance, residence, upbringing, lover experience, primary gaming type, and gaming history, no significant difference was found between LIGD and HIGD groups (*p >* 0.050). However, the HIGD group showed significantly longer gaming hours daily than the LIGD group (*t* = −9.562, *p <* 0.001).

**Table 1:** Basic information of the study sample.

Characteristics	LIGD (*N* = 52)	HIGD (*N* = 57)	*X*^2^/*t*	*p*
Age (years)	20.00 ± 1.55	19.74 ± 1.11	1.012	0.314
Gender (Male/Female, *N*)	35/17	39/18	0.015	0.901
Smoking (Yes/No, *N*)	7/45	3/54	2.193	0.189
Academic performance (Excellent/Others, *N*)	7/45	3/54	2.193	0.189
Residence (Urban/Rural, *N*)	22/30	26/31	0.121	0.847
Upbringing (Parents/Others, *N*)	51/1	53/4	1.612	0.366
Lover experience (Yes/No, *N*)	19/33	27/30	1.308	0.332
Primary gaming type (Roleplay/Others, *N*)	25/27	32/25	0.709	0.400
Gaming history (<2y/≥2y, *N*)	40/12	38/19	1.406	0.236
Gaming hours daily (hours)	1.56 ± 0.61	4.07 ± 1.88	−9.562	<0.001

### Group comparisons of psychological performances

As shown in Table 2. The HIGD group showed significantly increased BIS total (*t* = −2.875, *p =* 0.005), attention (*t* = −2.139, *p =* 0.035), motor (*t* = −2.017, *p =* 0.046), and non-planning (*t* = −2.171, *p =* 0.032) scores, but significantly decreased WLEIS emotion regulation score (*t* = 2.636, *p =* 0.010) and correct rate of eye tracking anti-saccade task (*t* = 2.294, *p =* 0.024) compared with LIGD group. Other indicators showed no significant difference between groups (*p >* 0.050).

**Table 2:** Differences in clinical psychological evaluations by scales and eye tracking anti-saccade task between groups.

Indicators	LIGD (*N* = 52)	HIGD (*N* = 57)	*X*^2^/*t*	*p*
BIS				
Total	56.27 ± 5.53	59.84 ± 7.38	−2.875	0.005
Attention	13.31 ± 2.31	14.25 ± 2.27	−2.139	0.035
Motor	18.44 ± 2.31	19.49 ± 3.03	−2.017	0.046
Non-planning	24.52 ± 3.62	26.11 ± 3.98	−2.171	0.032
WLEIS				
Total	59.52 ± 6.76	58.65 ± 8.12	0.605	0.547
Self-emotional appraisal	15.79 ± 2.14	16.00 ± 2.79	−0.441	0.660
Others’ emotional appraisal	13.62 ± 3.21	13.86 ± 3.04	−0.408	0.684
Emotion regulation	14.98 ± 2.40	13.54 ± 3.26	2.636	0.010
Use of emotion	15.13 ± 2.92	15.25 ± 3.06	−0.194	0.847
Anti-saccade task				
Correct rate (%)	42.46 ± 21.63	33.43 ± 19.45	2.294	0.024
Reaction time (s)	2.07 ± 2.88	2.15 ± 3.09	−1.300	0.196
Fixation count (times)	3.85 ± 0.67	5.14 ± 4.97	−1.943	0.057
Saccade count (times)	2.91 ± 0.69	4.23 ± 4.97	−1.985	0.052
Pupil size (mm)	2.06 ± 0.59	1.99 ± 0.79	0.522	0.603

### The correlation among BIS, WLEIS, and anti-saccade task

**Figure 1:**
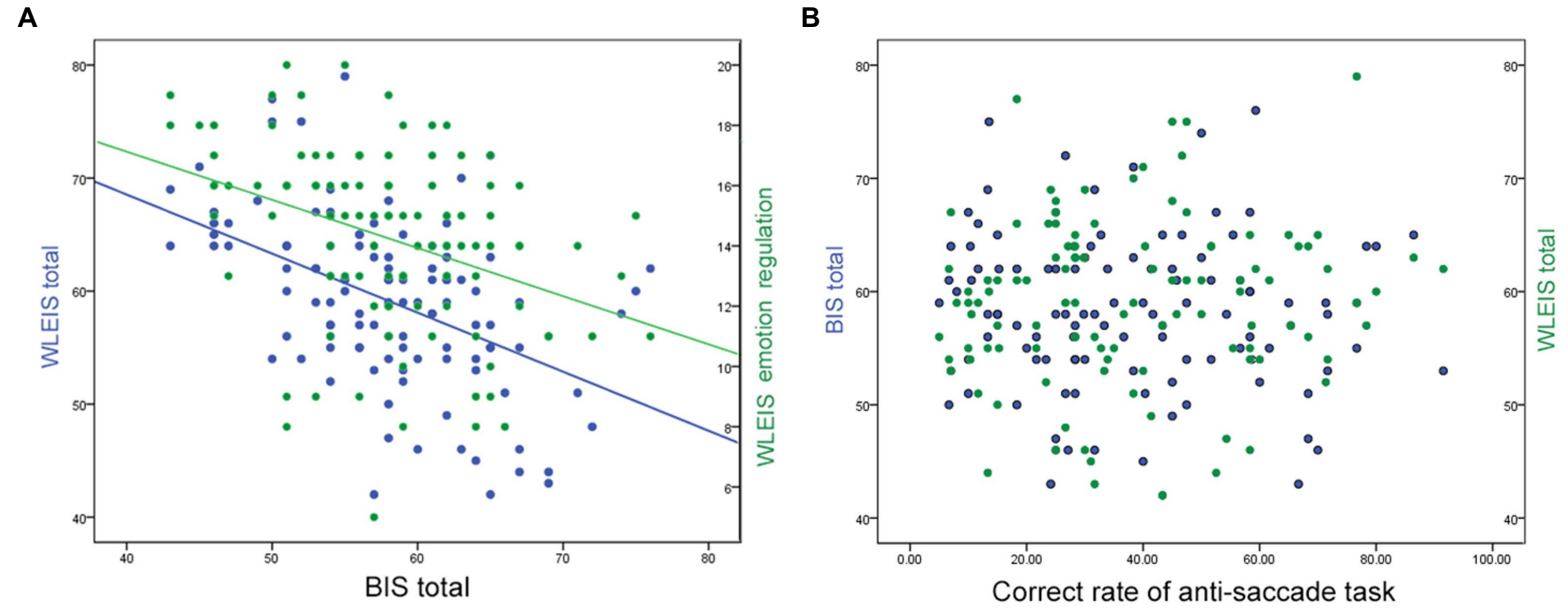
Correlations among BIS, WLEIS, and anti-saccade task. **(A)**, the correlation between BIS total and WLEIS total or emotion regulation scores; **(B)**, the correlation between the correct rate of anti-saccade task and BIS total or WLEIS total scores.

The BIS total score was negatively correlated with the WLEIS total (*r* = −0.473, *p <* 0.001) and WLEIS emotion regulation (*r* = −0.366, *p <* 0.001) scores (Figure 1A). No correlation was observed between anti-saccade results and BIS or WLEIS scores (*p >* 0.050, Figure 1B). Furthermore, the total score of BIS and WLEIS were positively correlated with their sub-scale scores (*p <* 0.050), respectively. The correct rate of anti-saccade task was negatively correlated with their reaction time (*r* = −0.701, *p <* 0.001), fixation count (*r* = −0.306, *p =* 0.001), and saccade count (*r* = −0.312, *p =* 0.001).

### Identifying potential marker for HIGD

**Figure 2:**
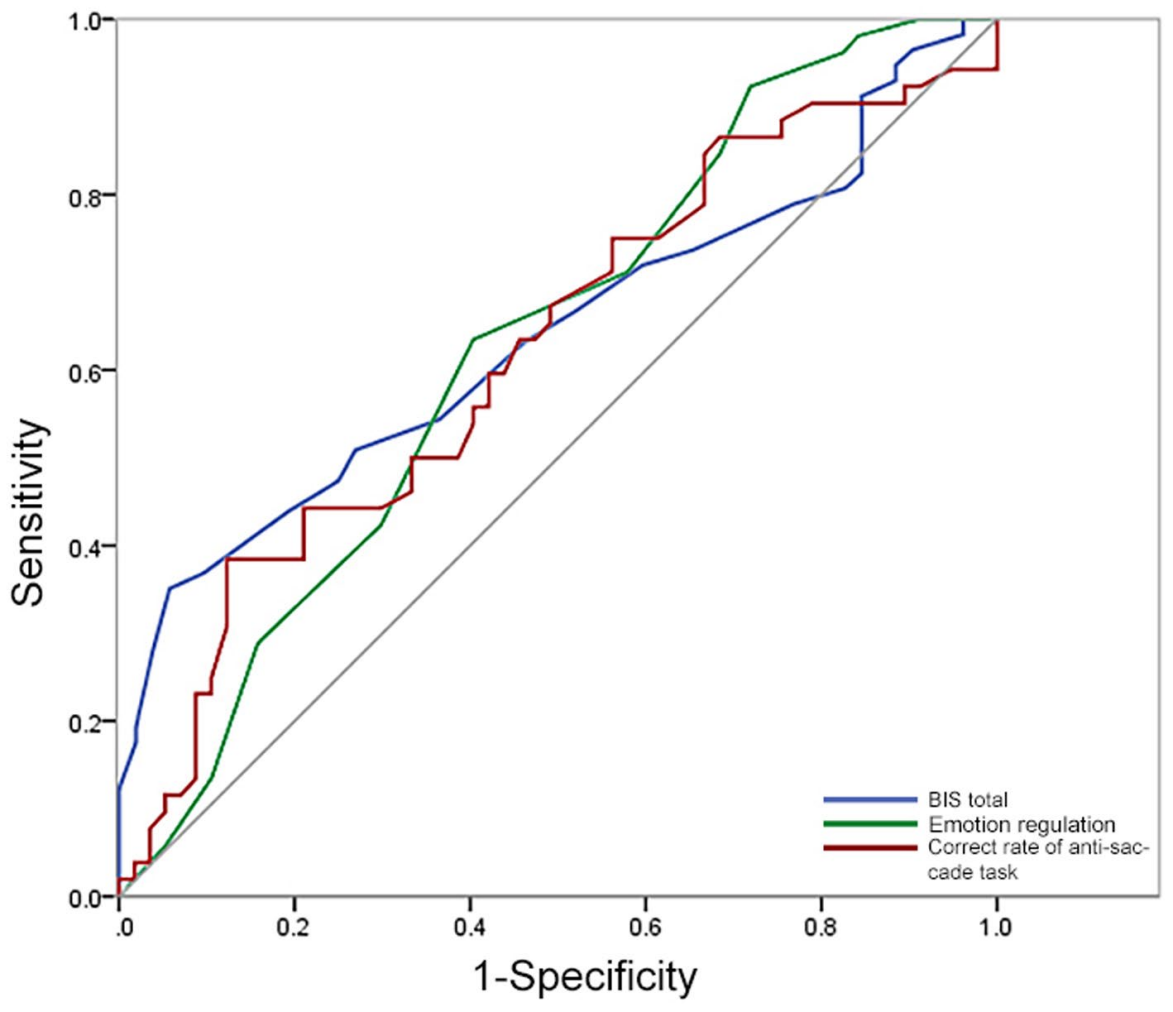
ROC of discriminating HIGD individuals from LIGD.

ROC analysis was used to identify the value of each indicator for discriminating HIGD from LIGD. The area under the curve (AUC), sensitivity, and specificity were 0.640, 35.1%, and 94.2% for BIS total, 0.628, 63.5%, and 59.6% for WLEIS emotion regulation, and 0.622, 38.5%, and 87.7% for correct rate of anti-saccade task (Table 3 and Figure 2), respectively. However, none of these indicators seems to be able to simultaneously discriminate HIGD from LIGD with satisfactory sensitivity and specificity.

**Table 3:** Discriminating HIGD from LIGD by ROC analyses.

Indicators	AUC (*95%CI*)	Cut-off value	Sensitivity	Specificity
BIS total	0.640 (0.536 ~ 0.744)	63.5	35.1%	94.2%
Emotion regulation (WLEIS)	0.628 (0.524 ~ 0.732)	14.5	63.5%	59.6%
Correct rate (Anti-saccade task)	0.622 (0.516 ~ 0.728)	53.4	38.5%	87.7%

**Figure 3:**
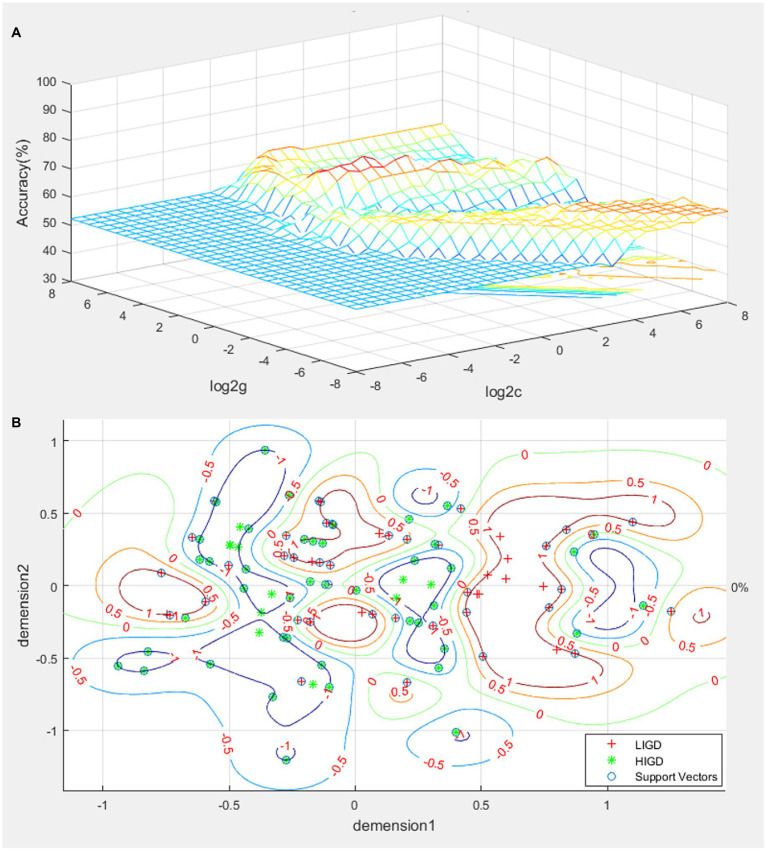
SVM of the combination of the correct rate of anti-saccade task and emotion regulation score. **(A)**, parameter optimization; **(B)**, visualization of classification.

Further, SVM analysis was used to identify the value of the combination of these indicators. The sensitivity, specificity, and accuracy of each combination for discriminating HIGD from LIGD are detailed in Table 4. A total of 95 of 109 subjects were correctly classified when a combination of the WLEIS emotion regulation score and correct rate of anti-saccade task with a sensitivity of 91.23%, a specificity of 82.69%, and an accuracy of 87.16%, which was the optimal combination with the highest accuracy (Table 4 and Figure 3). However, when combined with the daily gaming hours, the specificity could increase to 94.23%.

**Table 4:** Discriminating HIGD from LIGD by SVM analyses.

Indicator combination	Best c	Best g	Sensitivity	Specificity	Accuracy
BIS total + Emotion regulation (WLEIS)	0.35	0.25	59.65%	63.46%	61.47%
BIS total + Correct rate (Anti-saccade task)	1.41	5.66	75.43%	61.53%	68.81%
Emotion regulation (WLEIS) + Correct rate (Anti-saccade task)	2.83	16.00	91.23%	82.69%	87.16%
BIS total + Emotion regulation (WLEIS) + Correct rate (Anti-saccade task)	0.25	0.25	66.67%	55.77%	61.47%

### Logistic regression analysis

As shown in Table 5. Participants with higher gaming hours daily were 40.471 times more likely to be at the high risk than their counterparts (*Wald* = 14.308, *p <* 0.001, *95%CI*: 5.948 ~ 275.366). The daily gaming hours might be an independently associated factor for HIGD.

**Table 5:** Logistic regression analysis of developing to HIGD.

Variables	*SE*	*Wald*	*p*	*OR* (*95%CI*)
Age	0.344	0.177	0.674	0.865 (0.441 ~ 1.698)
Gender (Ref. female)	1.228	2.680	0.102	0.134 (0.012 ~ 1.487)
Smoking (Ref. no)	1.546	0.664	0.422	0.289 (0.014 ~ 5.989)
Academic performance (Ref. others)	4.951	0.371	0.543	0.049 (0.000 ~ 804.109)
Residence (Ref. rural)	0.982	0.219	0.640	0.632 (0.092 ~ 4.331)
Upbringing (Ref. others)	1.821	0.413	0.520	0.310 (0.009 ~ 11.011)
Lover experience (Ref. no)	0.912	0.324	0.569	1.681 (0.281 ~ 10.048)
Primary gaming type (Ref. others)	0.948	0.151	0.698	0.692 (0.108 ~ 4.438)
Gaming history (Ref. ≥2y)	0.901	3.159	0.076	0.202 (0.034 ~ 1.179)
Gaming hours daily	0.978	14.308	<0.001	40.471 (5.948 ~ 275.366)
BIS total	0.071	0.839	0.360	1.068 (0.928 ~ 1.228)
Emotion regulation (WLEIS)	0.181	0.005	0.942	1.013 (0.711 ~ 1.444)
Correct rate (Anti-saccade task)	0.025	0.139	0.709	1.009 (0.962 ~ 1.059)

## Discussion

The key finding of this study is that HIGD individuals exhibited a significantly higher BIS score, significantly lower WLEIS emotion regulation score, and significantly lower correct rate of eye tracking anti-saccade task when compared with LIGD. The BIS total score was negatively correlated with the WLEIS total and WLEIS emotion regulation scores. No correlation was observed between anti-saccade results and BIS or WLEIS scores. ROC analysis showed that none of these indicators of psychological performances could discriminate HIGD from LIGD with satisfactory sensitivity and specificity alone. However, SVM analysis revealed that the combination of the WLEIS emotion regulation score and the correct rate of anti-saccade task showed 91.23% sensitivity, 82.69% specificity, and 87.16% accuracy for discriminating HIGD from LIGD. In addition, participants with higher gaming hours daily were approximately 40-times more likely to be high risk than their counterparts.

Impulse control and emotion regulation were found to be abnormalities in patients with substance addiction (Fox et al., 2007) or non-substance addiction (Kruedelbach et al., 2006; Rogier and Velotti, 2018). In video gaming players, the scores in the behavioral inhibition system were significantly lower than the non-gaming player, suggesting that increasing impulsivity could mediate the maleficent impact on cognition (Jeong et al., 2020). A systematic review indicated that more significant IGD symptomology was associated with poorer emotion regulation (Marchica et al., 2019). However, the subscale of impulse control difficulties on the emotion regulation scale could predict the incidence of internet addiction (Tsai et al., 2020). In our study, the scores of BIS and WLEIS in HIGD were more abnormal than in LIGD, suggesting the impaired function of HIGD in impulse control and emotion regulation. In addition, the BIS score was negatively correlated with the WLEIS scores. Indeed, in some addictive disorders, impulse control and emotion regulation impairments often co-occur. However, their causal relationship also needs to be verified.

The anti-saccade task is a convenient and precise method to measure response inhibition (Munoz and Everling, 2004). So, the lowered correct rate of the anti-saccade task means impaired executive control function. For established patients with IGD, the correct rate of an anti-saccade task in the case of game-related images was significantly lower than in the other images, suggesting that patients with IGD were injured in inhibitory control (Kim et al., 2019). A meta-analysis that included 12 studies also got the same conclusion (Argyriou et al., 2017). Moreover, neural activity in some brain regions of adults with addiction, such as the front-parietal and the ventral attention network, was altered during response inhibition tasks using non-addiction-related stimuli (Qiu and Wang, 2021). In the present study, the HIGD population, which may contain unestablished IGD, had a significantly lower correct rate of an anti-saccade task than LIGD, suggesting an impaired response inhibition ability in HIGD. Hence, we inferred that game-related stimuli might directly affect the response inhibition function.

Previous studies are constantly looking for accurate indicators to assess whether an individual suffers from addiction (Kwako et al., 2018). ROC and SVM were used in the present study to assess the potential value of psychological performance in determining HIGD. Our ROC results indicated that the sensitivity or specificity of impulse control, emotion regulation, and response inhibition (individually) for discriminating the HIGD from LIGD are not high. An indicator with 0.6 or lower sensitivity or specificity seems to have low accuracy (Wang et al., 2018). However, the SVM analyses showed that the sensitivity, specificity, and accuracy were more than 0.8 in the combination of the abnormal psychological performances of the WLEIS emotion regulation score and the correct rate of the anti-saccade task for discriminating HIGD individuals from LIGD. Thus, we inferred that the combination of decreased emotion regulation and response inhibition abilities could be employed as a potential marker to identify HIGD individuals from LIGD.

The criterion of tolerance in DSM-5 for IGD refers to a need for increasing gaming time (King et al., 2017). Individuals spending ≤19 h online gaming per week more often felt out of control and lost important things less often than individuals spending >19 h online gaming per week (Wu et al., 2017). However, the thresholds of the hour spent gaming did not appear in any of the diagnostic criteria for IGD and its associated clinical scales. In the present study, our result showed that the daily gaming hours were significantly higher in HIGD than in LIGD. Further, the logistical regression analysis revealed that the participants with higher gaming hours daily were approximately 40-times more likely to have HIGD. The gaming hour is one of the essential associated factors for IGD (Rho et al., 2017).

Limitations also existed in the present study. Firstly, stratification of IGD risk mainly relies on the scales, but only one time of assessment for the subject might cause potential biases. Secondly, the study was a cross-sectional analysis and failed to observe the conversion between low and high risk. Thirdly, A larger sample is needed to verify the accuracy of the results. Despite the limitations, the present study infers those abnormal psychological performances exist in HIGD individuals, and a combination of emotion regulation and response inhibition could be applied as a potential marker to identify HIGD from LIGD. In clinical practice, the combination of worse emotion regulation (evaluated by scales) and poor response inhibition (evaluated by anti-saccade task) might contribute to the diagnosis of HIGD. Early detection of changes in these stable, comprehensive indicators will help reduce the incidence of such addiction. So, response inhibition and emotion management assessments could be considered routine risk screening items for IGD in clinical use.

## Conclusion

HIGD individuals have worse psychological performances of impulse control, emotion regulation, and response inhibition than LIGD. The decreased emotion regulation and response inhibition abilities could be employed as a potential marker to identify HIGD individuals from LIGD. Accurate identification of HIGD is the key to IGD prevention and treatment.

## Data availability statement

The original contributions presented in the study are included in the article/supplementary material, further inquiries can be directed to the corresponding authors.

## Ethics Statement

The studies involving human participants were reviewed and approved by The Ethics Committee of Chengdu Medical College. The patients/participants provided their written informed consent to participate in this study.

## Author contributions

All authors listed have made a substantial, direct, and intellectual contribution to the work, and approved it for publication.

## Funding

This study was supported by grants from the Disciplinary Construction Innovation Team Foundation of Chengdu Medical College (Grant No. CMC-XK-2105), Scientific Research Planning Project of Sichuan Psychological Association (Grant No. SCSXLXH2021013), and Research Foundation of Sichuan Applied Psychology Research Center (Grant No. CSXL-22223).

## Conflict of interest

The authors declare that the research was conducted in the absence of any commercial or financial relationships that could be construed as a potential conflict of interest.

## Publisher’s note

All claims expressed in this article are solely those of the authors and do not necessarily represent those of their affiliated organizations, or those of the publisher, the editors and the reviewers. Any product that may be evaluated in this article, or claim that may be made by its manufacturer, is not guaranteed or endorsed by the publisher.

## Footnotes

^1^ http://www.csie.ntu.edu.tw/~cjlin/libsvm
